# Intimate Partner Violence and Reproductive Coercion: Global Barriers to Women's Reproductive Control

**DOI:** 10.1371/journal.pmed.1001723

**Published:** 2014-09-16

**Authors:** Jay G. Silverman, Anita Raj

**Affiliations:** 1 Division of Global Public Health, Department of Medicine, University of California, San Diego School of Medicine, San Diego, California, United States of America; 2 Center on Gender Equity and Health, University of California, San Diego, California, United States of America

## Abstract

Jay Silverman and Anita Raj discuss the policies and interventions required to address the range of poor reproductive outcomes for women and adolescents, including loss of reproductive control, associated with intimate partner violence.

*Please see later in the article for the Editors' Summary*

**Summary Points**

- Intimate partner violence (IPV) is a major contributor to poor reproductive outcomes (e.g., adolescent and unintended pregnancy) among women and girls globally.
- To improve reproductive health, it is necessary that service provision goes beyond identification of women and girls affected by IPV to include identification of specific behaviors that reduce women and girls' control over their reproductive health, e.g., reproductive coercion, and assistance to reduce harm caused by these behaviors.
- In order to assist women and girls to mitigate the risks to their reproductive health caused by IPV and reproductive coercion, access to female-controlled contraceptive methods must be improved.
- In addition to assisting women and girls to improve their control over their reproductive health, reduction of IPV and reproductive coercion in the longer term requires ongoing and multiple-sector efforts to transform the social norms that maintain men's entitlement to control of women's and girls' bodies and their reproduction.

## Intimate Partner Violence (IPV) and Reproductive Control

According to a recent multi-country study by the World Health Organization (WHO), between 23% and 49% of females aged 15 years and older are subjected to IPV across a majority of nations [1], underscoring the scope of this major global health and human rights issue. Two decades of research across multiple global regions have documented that IPV is associated with poor reproductive outcomes for women and girls; women experiencing IPV are twice as likely to have a male partner refuse to use contraception [1],[2] and to report unintended pregnancy [2]–[6], and up to three times more likely to give birth as an adolescent compared to those not experiencing such violence [7]. The loss of reproductive control accompanying IPV is further indicated by the significantly greater likelihood of abused women to have five or more births than those with a nonviolent male partner [1].

Importantly, women who have partners that are abusive are twice as likely to have had an induced abortion and three times as likely to have had experienced multiple abortions than women who do not report IPV [1],[3],[4],[8]. Although these associations logically follow from the higher likelihood of unintended pregnancy, because abortion is too often conducted without required hygiene and by individuals without adequate medical training (particularly in national contexts in which abortion is legally restricted) [9], it is one of the three leading causes of global maternal mortality, with more than one in eight maternal deaths globally due to unsafe abortion [9]. Thus, IPV places women at greater risk of maternal death by increasing their risk for unsafe abortion, with abusive male partners also observed to use coercion to control women's decisions regarding abortion [10]; in the single study of abortion coercion, men reporting perpetrating IPV were more likely to attempt to coerce a pregnant female partner both into having an abortion against her will and continuing a pregnancy that she wanted to terminate [8].

Given the high risk of unintended pregnancy and need for abortion based on IPV, the WHO recommends that key services be made available to women and girls identified as experiencing IPV, including both emergency contraception and safe abortion [11]. These guidelines also recommend that identification and support of abused women be conducted within the context of reproductive health services globally [11]. Although integration of IPV screening and counseling have been implemented across high-, middle-, and low-income countries [12], to date, no such model has demonstrated a significant reduction in risk for unintended pregnancy or other adverse reproductive health outcomes. One likely barrier to such effective innovation is that programs may need to not only identify women and girls affected by IPV but also identify and target the specific behavioral mechanisms underlying the associations of IPV with poor reproductive outcomes, i.e., those behaviors directly related to women and girls' lack of reproductive control [10].

## Reproductive Coercion

One potential behavioral mechanism for the consistent associations of IPV and poor reproductive health that has been suggested by recent research is reproductive coercion [6],[10]. This phenomenon is described in a recent position paper from the American College of Obstetrics and Gynecology as behavior that interferes with contraception use and pregnancy in ways that reduce female control over reproductive decisions, including pregnancy coercion and contraceptive sabotage (see Box 1) [13]. Pregnancy coercion describes forms of male partner behavior that are intended to erode a female partner's ability to resist complying with a male partner's wishes that she become pregnant or that she continue or terminate a pregnancy [6],[10],[13]. Such coercion may include threats or actual violence against a female partner to force her to comply with demands that she become pregnant (e.g., blocking access to family planning services) or that she continue or terminate a pregnancy (e.g., blocking access to abortion services, compelling a female partner to abort a pregnancy under duress, or injuring a female partner in order to induce abortion) [6],[10],[13]. Contraceptive sabotage may include hiding, withholding, destroying, or removing female-controlled contraceptives (e.g., oral contraceptives, intrauterine devices, contraceptive patches) or deliberately breaking or removing a condom during sex or failing to withdraw in an attempt to promote pregnancy despite a female partner's wishes to prevent pregnancy [6],[10],[13]. The inclusion of threats of violence and force in reproductive coercion builds on the concept of “symbolic violence,” which describes the likely independent effects of threats of violence, beyond those of the violence itself [14],[15].

**Box 1. Definitions of Reproductive Coercion**

**Reproductive coercion** consists of behaviors that directly interfere with contraception and pregnancy, reducing female reproductive autonomy. The two forms of reproductive coercion are pregnancy coercion and contraception sabotage.

**Pregnancy coercion** includes behaviors to coerce compliance with a male partner's desire that a woman or girl become pregnant, or his desire that she continue or terminate a pregnancy against her will. These include threats or actual violence to force her to comply with demands that she become pregnant or terminate a pregnancy, blocking access to family planning services, and preventing access to abortion services or being made to undergo abortion under duress. [6],[10],[13]

**Contraception sabotage** relates to partner behaviors that purposely interfere with a woman's attempts to prevent pregnancy including hiding, withholding, destroying, or removing female-controlled contraceptives (e.g., oral contraceptives, intrauterine devices, contraceptive patches); deliberately breaking or removing a condom during sex; and failing to withdraw in an attempt to promote pregnancy despite a female partner's wishes to prevent pregnancy [6],[10],[13].

Findings of several recent studies indicate that women and girls who report physical and sexual IPV are significantly more likely to also experience reproductive coercion from male partners [6],[16], and the single study of these issues among men found that those who report perpetrating IPV are three times more likely than non-abusive men to perpetrate abortion coercion, i.e., coercion to compel a pregnant female partner to either terminate or continue a pregnancy against her will [8]. Furthermore, this research also indicates that reproductive coercion predicts unintended pregnancy independent of the effects of IPV, as well as interacting with IPV to heighten risk for unintended pregnancy beyond that seen for IPV alone [6]. These findings and the concept of reproductive coercion are consistent with the body of literature demonstrating that norms of gender inequity are associated with poor reproductive heath outcomes independent of actual experiences of IPV [17],[18]. Reproductive coercion builds upon these concepts and research in that it includes nonviolent behaviors that represent mechanisms for which IPV is a marker and that appear to underlie the observed associations between IPV and poor reproductive health outcomes.

Recent studies in Côte d'Ivoire and Jordan indicate that, not only do the concept of reproductive coercion and the association between these behaviors and poor reproductive health appear to extend to low- and middle-income countries, but also that potential perpetrators of reproductive coercion include in-laws [19],[20]. Moving beyond epidemiologic studies, recent research in the United States has described the development and evaluation of a reproductive health clinic-based intervention to reduce reproductive coercion in order to reduce unintended pregnancy [21]. Intervention elements focus on identification of reproductive coercion and provision of harm reduction strategies, i.e., behaviors to minimize the risk for unintended pregnancy in the context of reproductive coercion (e.g., utilizing contraceptive methods that are difficult for a male partner to detect or block such as injectable forms and intrauterine devices, or strategies to reduce detection of current forms of contraception) in order to to assist women in reducing the impact of this coercion [13],[21]. This approach is incorporated into standard family planning practice in the US and delivered by existing family planning paraprofessionals in order to maximize sustainability and scalability [13]. Evidence from the single published randomized controlled trial (RCT) of this model indicates that receipt of this brief intervention leads to reductions in both experiences of reproductive coercion and IPV [21]. Although a larger and longer-term RCT of this model capable of assessing effects on unintended pregnancy is required, based on the promise of this approach, the American College of Obstetricians and Gynecologists has recommended that reproductive coercion be identified and addressed within US reproductive health services for women and girls [13].

One criticism of this type of approach may be the apparent burden it places on the women and girls who are likely to have limited control of the behavior of violent and coercive male partners. In addition, relative to US women's options regarding rejection of a male partner, the greater social and economic limitations faced by women in many low- and middle-income countries may make it less likely that they would able to successfully implement strategies to reduce sexual coercion and violence. However, a recent randomized controlled study conducted in India demonstrated that harm reduction counseling provided to women who reported having husbands who were abusive and/or drank heavily resulted in significant reduction in subsequent exposure to spousal sexual violence [22].

In addition to the previously mentioned studies in West Africa and the Middle East, the relevance of this intervention approach to low- and middle-income countries is supported by findings of a recent regional study of the association of IPV to contraceptive strategies indicative of women's responses to reproductive coercion in the South Asian nations of Bangladesh, India, and Nepal [23]. Findings indicate that women who report sexual IPV victimization in these national contexts are more likely to report recent use of any modern contraception (not including sterilization) [23]. However, when use of individual forms of contraception is examined, women abused by male partners are found to be less likely to use the form typically controlled by a male partner, male condoms, but more likely to have used forms of contraception that are female-controlled (e.g., oral contraceptive pills) [23]. These findings are consistent with both the literature and clinical recommendations regarding reproductive coercion and partner violence, in that abusive men were found to be less likely to use condoms, and women coping with such abuse may be more likely to rely on female-controlled methods to minimize their increased risk for unwanted pregnancy [13],[21]. Importantly, these findings also point to the need to prioritize increasing access to female-controlled contraceptive methods [24]; in India, a country with tremendous unmet need regarding spacing contraception [25], major forms of modern long-acting hormonal contraceptives (e.g., those that are injectable) remain unavailable within the public health care sector.

## Policy Implications

Addressing the range of poor reproductive outcomes associated with IPV and related nonviolent forms of male reproductive coercion will require development of interventions and policies at multiple levels (e.g., national health care system, clinic, community, family, relationship) adapted to multiple cultural and geographic contexts. Also, while approaches such as addressing reproductive coercion may hold great promise regarding minimizing the impact of IPV on women's control of family planning [13], it must be recognized that reduction of IPV and reproductive coercion in the longer term will require ongoing and multiple-sector efforts to transform the social norms that maintain men's entitlement to control of women's and girls' bodies and their reproductive health (see Box 2) [8].

**Box 2. Recommendations**

1. Reproductive health providers should be trained to provide confidential education and counseling to assist women in clarifying their own family planning goals regarding timing, number, and spacing of pregnancies, as well as to help them reduce the partner-related barriers they may face in implementing these plans (i.e., reproductive coercion).
2. A full range of contraceptive methods should be made broadly available at little or no cost, particularly those which are most within the control of women (e.g., injectable and intrauterine forms) in order to increase women's ability to space and prevent pregnancies and to maintain control over these decisions.
3. Effective, sustainable, and scalable programs to address attitudes and norms that maintain abusive and controlling behaviors among men and boys should be considered for implementation within multiple sectors in order to reduce acceptability of men's perpetration of IPV and related domination of family planning decisions.

At the health system level, availability of a broad range of contraceptive methods, particularly those which are most within the control of women (e.g., injectable and intrauterine forms) at no or low cost is critical to increasing women's ability to space and prevent pregnancies and to safely control these decisions [24],[25]. Along with this expansion, policies must be developed or strengthened that ensure that these methods are only provided with women's full and informed consent to reduce the potential for women being coerced to reduce their fertility, as has been practiced previously in multiple nations via forced sterilization [26]. Health systems should also provide education and access to medical abortion [27]. Such access is critical to improve women's control of decisions regarding termination of a pregnancy, particularly for the large segment of women experiencing IPV [8],[27]. Barriers faced by an abused woman in procuring an abortion likely include travel to surgical venues, easy detection of surgical intervention, and required recovery periods, as these elements are likely impossible to obscure from a male partner. Medical abortion, in contrast, may allow women to access abortion services at local, nonsurgical venues and to obtain an abortion for which the observable sequelae are similar to those for a miscarriage [27], thus, likely reducing the risk of retaliation from an abusive partner who has sought to compel her to continue the pregnancy.

At the clinic level, reproductive health providers must be trained to provide confidential education and counseling to assist women in clarifying their own family planning goals regarding timing, number, and spacing of pregnancies, as well as the barriers they may be facing in implementing this plan (i.e., reproductive coercion) [13]; this information is vital in enabling providers to educate and guide women in choosing the most safe and appropriate method of contraception in the context of such challenges to their reproductive autonomy [21]. Furthermore, cases of violence from male partners will be disclosed when counseling of this type is provided in a confidential and respectful manner [10], and reproductive health providers should be educated regarding the nature and availability of services for abused women so as to competently provide information and referrals to such services [11],[13].

At the community level, information must be shared with leaders from multiple sectors (e.g., teachers, coaches, clergy, business owners, elders, and other civic leaders) such that they understand the many costs of IPV to their communities. Based on this understanding, a collective commitment should be made to change social norms that support men's perpetration of IPV [24] and related domination of family planning decisions. Making such changes will likely involve work within schools, athletic teams, religious organizations, local governing bodies, businesses, and service providers, including those who may be counseling men on contraception (e.g., private providers of sexual health remedies for men) [28]. Notable recent examples from South Africa and India of programs that address attitudes and norms that maintain abusive and controlling behaviors among men and boys [29]–[31] and other community-based approaches of this kind should be should carefully considered for efficacy, adaptability, and scaling at the population level.

The high prevalence of IPV across regions, and the related reductions in female reproductive autonomy in multiple cultural and geographic contexts, are major drivers of unintended pregnancies and need for abortion among women and girls. Thus, empowering women and girls to increase control of their reproductive health via brief and sustainable health service–based programs to reduce male partner coercion related to contraception and pregnancy, while simultaneously targeting the social norms that maintain these gendered male behaviors and their broad acceptance, may be a potent approach to improving the reproductive health of women and girls globally.
